# Causes of Down syndrome regression disorder: a scoping review

**DOI:** 10.1590/1980-5764-DN-2024-0233

**Published:** 2025-07-18

**Authors:** Manuelle Maria Pereira Natividade, Adson José Moreira, Lívia Silva Nassif, Bárbara Rodrigues Alvernaz dos Santos, Marcus Carvalho Borin, Juliana Alvares-Teodoro, Francisco de Assis Acurcio, Augusto Afonso Guerra

**Affiliations:** 1 Universidade Federal de Minas Gerais, Faculdade de Farmácia, Departamento de Farmácia Social, Belo Horizonte MG, Brazil.; 2 Universidade Federal de Minas Gerais, Faculdade de Farmácia, Belo Horizonte MG, Brazil.

**Keywords:** Autoimmunity, Down Syndrome, Immune System, Neurocognitive Disorders, Stress, Psychological, Autoimunidade, Síndrome de Down, Sistema Imunitário, Transtornos Neurocognitivos, Estresse Psicológico

## Abstract

Down syndrome regression disorder (DSRD) is characterized by an acute or subacute neurocognitive regression that severely impacts the autonomy and quality of life of individuals with Down syndrome (DS). Despite its growing recognition, understanding of the condition remains limited, particularly regarding its etiology and the factors contributing to its development.

**Objective:**

The aim of this study was to systematically map the available evidence regarding the potential causes of DSRD and the factors that may contribute to its development.

**Methods:**

Following the Joanna Briggs Institute (JBI) methodology for scoping reviews, a comprehensive three-step search strategy was conducted across MEDLINE (PubMed), Embase, Cochrane, and Lilacs. Studies published in any language were considered, with no restrictions on publication date.

**Results:**

In total, 14 studies met the eligibility criteria. The findings consistently point to chronic autoimmunity and immune dysregulation as potential causes of DSRD. Additionally, the contribution of genetic variants associated with the type 1 interferon inflammatory response has been suggested. Finally, the role of psychosocial and environmental stressors was highlighted, as these are considered potential triggers that precede the onset of DSRD manifestations.

**Conclusion:**

The hypothesis that DSRD is a multifactorial condition seems reasonable. Nevertheless, the immune system may play a central role in its development, as the identified causes converge toward a neuroinflammatory process. Furthermore, the contribution of genetic variants associated with the inflammatory response and the role of psychosocial stressors as triggers for DSRD also appear plausible.

## RESUMO

O transtorno de regressão da síndrome de Down (DSRD) se caracteriza por ser um quadro agudo ou subagudo de regressão neurocognitiva que impacta sobremaneira a autonomia e a qualidade de vida das pessoas com síndrome de Down. A despeito do seu crescente reconhecimento, a compreensão dessa condição ainda é limitada, especialmente no que diz respeito à sua etiologia e aos fatores que contribuem para o seu desenvolvimento.

**Objetivo:**

Mapear sistematicamente as evidências disponíveis acerca das possíveis causas do DSRD e dos fatores que podem contribuir para o seu desenvolvimento.

**Métodos:**

Seguindo a metodologia do Instituto Joanna Briggs para revisões de escopo, foi realizada uma estratégia de busca abrangente em três etapas nas bases MEDLINE (PubMed), Embase, Cochrane e Lilacs. Estudos publicados em qualquer idioma foram considerados, sem restrições quanto à data de publicação.

**Resultados:**

Quatorze estudos atenderam aos critérios de elegibilidade. Os achados apontaram consistentemente para a autoimunidade crônica e a desregulação imunológica como potenciais causas do DSRD. Além disso, sugeriu-se a contribuição de variantes genéticas associadas à resposta inflamatória do interferon tipo 1. Por fim, evidenciou-se o papel dos estressores psicossociais ou ambientais, considerados potenciais gatilhos que precedem o início das manifestações do DSRD.

**Conclusão:**

A hipótese de que o DSRD é uma condição multifatorial parece razoável. Não obstante, o sistema imunológico pode desempenhar um papel central no seu desenvolvimento, uma vez que as causas identificadas convergem para um processo neuroinflamatório. Ademais, a contribuição de variantes genéticas associadas à resposta inflamatória e a atuação dos estressores psicossociais como gatilhos para o DSRD também parecem plausíveis.

## INTRODUCTION

Down syndrome (DS) occurs in 1 out of every 800–1,000 live births, with more than 214,000 individuals with DS living in the United States and 417,000 in Europe. It is the most common genetic cause of intellectual disability worldwide, and neurological and psychiatric conditions in this population are well-established. However, there is still limited knowledge regarding a specific health condition af fecting this group, whose number of reported cases has increased in the past decade: Down syndrome regression disorder (DSRD)^1^ .

DSRD is often a severe condition that significantly impacts the autonomy of individuals with DS, as well as the quality of life of both the individuals and their caregivers. It presents as an acute or subacute neurocognitive regression, occurring independently of the individual’s cognitive performance^1-3^. DSRD is characterized by the loss or deterioration of previously acquired developmental milestones in areas such as language, communication, cognition, executive function, daily activities, and behavioral and adaptive skills. Neuropsychiatric manifestations vary and may include bradykinesia, catatonia, agitation, insomnia, mutism, hallucinations, and depersonalization^1,2,4,5^. 

Regarding the natural course of DSRD, an acute phase appears to last approximately 6 months, followed by a chronic phase characterized by a variable recovery of lost abilities, which differs from dementia, where pro gression without recovery is the expected outcome. Two studies reported that 58% of individuals with DSRD experienced partial or total recovery, 35% stabilized (in these cases complete recovery to the premorbid baseline condition appears to be infrequent), and finally, 7% of patients showed additional worsening^4^ .

The exact prevalence of DSRD remains unknown, but existing reports suggest that the disorder predom inantly affects individuals between the ages of 10 and 30, with a female-to-male ratio of approximately 2:1 ^3^ . Therapeutic interventions have primarily focused on managing the symptoms, incorporating low-dose psy chotropic medications such as anxiolytics, antipsychot ics, and antidepressants. In addition, immunotherapy has shown promise, and electroconvulsive therapy has been used in more severe cases. These treatments aim to mitigate the neuropsychiatric symptoms that signifi cantly impair the quality of life for both patients and their caregivers^4,5^.

Given the absence of definitive biomarkers, DSRD is considered a diagnosis of exclusion, highlighting the importance of a multidisciplinary approach. Clinical recommendations for diagnosing DSRD were pub lished in 2022, following an international consensus of experts in DS. According to the consensus, the sudden onset (within 12 weeks) of new neurological and/or psychiatric changes in previously healthy in dividuals with DS should raise suspicion. The experts identified 28 potential changes and categorized them into eight groups:

- altered mental state or behavioral dysregulation;
- cognitive decline;
- developmental regression with or without new manifestations of autism;
- new focal neurological deficits or seizures;
- insomnia or circadian rhythm disturbances;
- language deficits;
- movement disorders (excluding tics);
- psychiatric symptoms.

A diagnosis is considered possible when the patient presents changes in more than three groups and probable when changes are observed in more than six groups^1^ . 

 As an emerging issue, knowledge about DSRD remains fragmented, with significant gaps yet to be addressed. One of these gaps involves understanding the causes and determining factors of the disorder. So far, existing hypotheses are largely drawn from the etiology of regression in other neurodevelopmental disorders, suggesting genetic, neurological, immunological, and psychiatric causes^3^ . Furthermore, most studies conducted on DSRD to date are limited to small data sets and case series^1^ . 

 A preliminary search of the Open Science Framework (OSF), PROSPERO, PubMed, Cochrane, and JBI platforms was conducted, and no current or ongoing scoping reviews on the causes of DSRD were identified. Given the importance of understanding the etiology for developing guidelines on diagnosis and treatment, this review seeks to map the available evidence in the literature regarding the possible causes of DSRD and the factors contributing to its development. 

## METHODS

This scoping review was conducted following the Joanna Briggs Institute (JBI)^6^ methodology and was registered on the OSF under the DOI 10.17605/OSF.IO/47Q9E. 

### Review question

The question "What are the possible causes of Down syndrome regression disorder (DSRD)?" was used to develop the research methodology based on the PCC (Population, Concept, and Context) framework.

### Inclusion criteria

#### Participants

 The target population for this review consisted of individuals with DS who had a confirmed diagnosis of DSRD. 

#### Concept

 The core concept of this scoping review was the "Etiology of Down syndrome regression disorder (DSRD)." The review aimed to identify potential biological, psychological, and social factors that may contribute to the development of this condition. 

#### Context

 This review included studies without restrictions based on geographical location, ethnicity, age, or sex, focusing on individuals with DS who had been diagnosed with DSRD. 

#### Types of sources

 Eligible sources included studies that explored potential causes or contributing factors to the onset of DSRD. Both descriptive observational studies (case series and individual case reports) and analytical observational studies (non-concurrent cohorts and case–control studies) were included, alongside systematic reviews, narrative reviews, and expert consensus guidelines. 

### Search strategy

 A three-step search strategy was employed for this review. First, an initial limited search of MEDLINE (PubMed) and Embase was conducted to identify relevant articles on the topic. The keywords and index terms used in the titles and abstracts of these articles were then utilized to develop a comprehensive search strategy. This strategy, which included keywords and identified index terms, was applied in a secondary search across databases such as MEDLINE (PubMed), Embase, Cochrane, and Lilacs, with appropriate adaptations for each database (Table 1). Finally, a tertiary search was conducted by screening the reference lists of the included sources to identify any additional relevant studies. The search strategy was conducted on August 17, 2024. 

**Table 1:** Search strategy across databases.

Database	Search strategy	N
MEDLINE (Pubmed)	("Down Syndrome"[Mesh] OR Syndrome, Down OR Down’s Syndrome OR Downs Syndrome OR Syndrome, Down’s OR Mongolism OR Trisomy 21 OR Trisomy G OR 47,XX,+21 OR 47,XY,+21 OR Down Syndrome, Partial Trisomy 21 OR Partial Trisomy 21 Down Syndrome OR Trisomy 21, Meiotic Nondisjunction OR Trisomy 21, Mitotic Nondisjunction) AND (((regression disorder[Title/Abstract]) OR (disintegrative disorder[Title/Abstract])) OR (unexplained regression[Title/Abstract]))	36
Embase	"down syndrome":ti,ab,kw AND "regression disorder":ti,ab,kw OR "disintegrative disorder":ti,ab,kw OR "unexplained regression":ti,ab,kw	190
Cochrane	("Down syndrome"):ti,ab,kw AND (regression disorder):ti,ab,kw OR (disintegrative disorder):ti,ab,kw OR (unexplained regression):ti,ab,kw	8
Lilacs	("down syndrome") AND ("regression disorder")	17
	("down syndrome") AND ("disintegrative disorder")	11
	("down syndrome") AND ("unexplained regression")	11

### Study/source of evidence selection

 Following the search, the selected studies were uploaded to the Rayyan^7^ QCRI platform, and duplicates were removed. The initial screening was performed by assessing the titles and abstracts according to the predefined inclusion criteria, with two independent reviewers conducting the evaluation. Potentially relevant studies were retrieved, and their full texts were reviewed by two independent reviewers, adhering to the inclusion requirements. Studies published in any language were included, with no restrictions on publication date. The reasons for excluding sources of evidence were documented and reported in the PRISMA^8^ flowchart. Any discrepancies identified between the reviewers at each stage of the selection process were resolved by a third, independent reviewer. The studies included in the scoping review were assessed using the JBI Critical Appraisal Tools^9^ , with specific checklists applied according to the study design. 

### Data extraction

 Data were extracted from the included articles by two independent reviewers, and the information was recorded in a spreadsheet (Microsoft Excel). The data extraction form included details on the year of publication, title, authorship, study objective, study type, number of participants, and key findings regarding causes and triggers associated with DSRD. Any discrepancies between the reviewers were resolved through discussion or by consulting a third reviewer. 

## RESULTS

**Figure 1:**
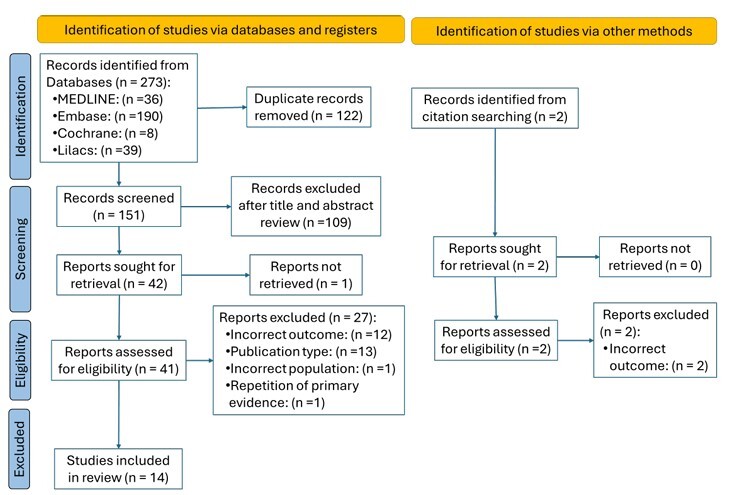
PRISMA flow diagram.

Source: Adapted from Page et al.^8^ .

 The results of the search and study selection process are outlined in Figure 1. After applying the complete search strategy, 273 publications were retrieved from databases such as MEDLINE (PubMed), Embase, Cochrane, and Lilacs. Of these, 122 duplicates were removed, leaving 151 studies eligible for title and abstract screening. After this screening, 41 publications remained for full-text review. Of these, 27 were excluded: 13 for publication type (abstracts and letters to the editor), 12 for incorrect outcomes (as they did not meet the PCC question criteria in terms of context or concept), one for an incorrect population, and one for duplicate primary evidence. 

A manual search was conducted by reviewing the reference lists of the included sources of evidence. In this process, two additional publications were identified, but both were excluded after full-text review due to incorrect outcomes. In the end, 14 studies met the eligibility criteria, and after being assessed using the JBI Critical Appraisal Tools^9^ were included in this review (Supplementary Material). 

 These comprised five case-control studies, three cohort studies, three case series, two case reports, and one systematic review. 

Table 2 presents the extracted data from the selected studies, including year of publication, title, author ship, objective, study type, number of participants, and key findings regarding causes and triggers associated with DSRD. 

**Table 2:** Description of the studies included in the review.

N	Year	Title	Authorship	Objective	Study type	Number of participants	Key findings on causes and triggers
1	2015	Down syndrome disintegrative disorder: new-onset autistic regression, dementia, and insomnia in older children and adolescents with Down syndrome	Worley et al.^10^	To investigate the occurrence of new-onset autistic regression, dementia, and insomnia in children and adolescents with Down syndrome.	Case-control	11 (DSRD) and 21 (non DSRD)	The seropositivity rate was higher in individuals with DSRD than in those without DSRD.
2	2017	Acute regression in young people with Down syndrome.	Mircher et al.^11^	To share the authors’ experience with the report of 30 cases of regression in young DS who were followed longitudinally.	Cohort	30	The presence of a stressful event has been reported in most cases of DSRD.
3	2019	Immunotherapy in selected patients with Down syndrome disintegrative disorder.	Cardinale et al.^12^	Evaluate the response to immunotherapy in a small cohort of patients with DSRD who had evidence of autoimmunity.	Case series	4	All individuals demonstrated evidence of autoimmunity, testing positive for one, two, or four autoantibodies.
4	2020	Unexplained regression in Down syndrome: 35 cases from an international Down syndrome database.	Santoro et al.^13^	Describe cases of DSRD and compare them with age- and sex-matched Down syndrome patients.	Case-control	35 (DSRD) and 35 (non DSRD)	Individuals with DSRD had a higher average number of stressful events.
5	2021	A systematic review of unexplained early regression in adolescents and adults with Down syndrome.	Walpert et al.^14^	Identify patterns of symptomatology, potential trigger events, prognosis, treatments, and outcomes surrounding unexplained regression in adolescents and young adults with Down syndrome.	Systematic review	Not applicable	DSRD can best be considered as a stress triggered condition that occurs in people with Down syndrome who have some genetic or acquired vulnerability.
6	2021	Case report: improvement following immunotherapy in an individual with seronegative Down syndrome disintegrative disorder.	Hart et al.^15^	Present the case of an 8-year-old girl with DSRD without evidence of autoimmunity.	Case report	1	The patient showed marked improvement in symptoms after treatment with IVIg and steroids.
7	2022	Is developmental regression in Down syndrome linked to life stressors?	Sargado et al.^16^	Understand the role of psychosocial stressors in DSRD.	Case series	14	All individuals experienced at least one and often several psychosocial stressors in the year preceding DSRD.
8	2022	Evidence of neuroinflammation and immunotherapy responsiveness in individuals with Down syndrome regression disorder.	Santoro et al.^3^	Investigate the potential role of neurologic and neuroimmunologic dysfunction in persons with DSRD and whether the presence of abnormalities dictates response to particular therapeutic interventions.	Case-control	72 (DSRD) and 1217 (non-DSRD)	The study reports multiple neurodiagnostic study abnormalities in nearly half of individuals with DSRD and preferential response to immunotherapy.
9	2022	Abnormal weight loss in an adolescent female with Down syndrome.	Garcia and Litra^17^	Report the case of a patient with Down syndrome who exhibited weight loss, altered mental status, and loss of functional skills over a period of 1 month.	Case report	1	The patient exhibited positive results for anti-TPO and thyroid-stimulating immunoglobulin (TSI) antibody. Changing medications and restarting high school were considered triggers.
10	2023	Immunotherapy responsiveness and risk of relapse in Down syndrome regression disorder.	Santoro et al.^5^	Investigate possible demographic, laboratory, and clinical factors associated with the response to IVIg immunotherapy and to assess the likelihood of gradually tapering the treatment successfully once symptom improvement has been achieved.	Cohort	82	Individuals with a history of autoimmunity or neurodiagnostic abnormalities were more likely to experience a clinical relapse upon wean of immunotherapy.
11	2023	Adverse childhood experiences and the development of Down syndrome regression disorder.	Wang et al.^18^	Evaluate whether adverse childhood experiences (ACEs) were more prevalent in children with DRSD than in those with Down syndrome alone.	Case-control	159 (DSRD) and 178 (non-DSRD)	Three or more ACEs was more prevalent in individuals with DSRD.
12	2023	Down syndrome regression disorder, a case series: clinical characterization and therapeutic approaches.	Bonne et al. ^2^	Describe the DSRD, discuss its etiologies and propose therapeutic strategies.	Case series	4	All patients presented positive autoimmune work ups and they responded to corticosteroid and anti inflammatory.
13	2023	Alternative diagnoses in the work up of Down syndrome regression disorder.	Santoro et al.^19^	Review non-DSRD diagnoses at a quaternary medical center specializing in the diagnosis of DSRD and compare clinical characteristics between those diagnosed with DSRD and those with non-DSRD diagnoses.	Case-control	212 (DSRD) and 54 (non DSRD)	A significant difference for the history of autoimmune disease and the presence of serum cytokine abnormalities was observed between DSRD and non-DSRD.
14	2024	De novo variants in immune regulatory genes in Down syndrome regression disorder.	Jafarpour et al.^20^	Evaluate contribution of rare variants within coding regions of genes related to immune regulation to DSRD.	Cohort	41 (DSRD with exome sequencing) and 306 (DSRD without exome sequencing)	Heterozygous de novo variants in immune regulation genes were identified in individuals with DSRD.

## DISCUSSION

 This scoping review synthesized the available scientific evidence on the potential causes of DSRD and the factors that may contribute to its development. Across the reviewed studies, chronic autoimmunity and immune dysregulation emerged as leading hypotheses. Additionally, genetic variants related to the type 1 interferon inflammatory response were implicated as potential contributors. Psychosocial and environmental stressors were also identified as possible triggers for DSRD, reinforcing the multifactorial nature of this condition. While the evidence suggests a complex interplay of genetic, immunological, and environmental factors, the scarcity of large-scale studies underscores the need for further research to better elucidate these mechanisms. 

 It is well-established that DS is associated with elevated levels of pro-inflammatory cytokines and an increased risk for autoimmune diseases^12,15^. Considering the potential immunological etiology of DSRD, one study presented a series of four cases in which all individuals exhibited evidence of autoimmunity. Patients tested positive for one, two, or four auto antibodies, including three thyroid-related antibodies: anti-thyroid peroxidase (anti-TPO), anti-thyroid-stimulating hormone (anti-TSH), and anti-microsomal antibodies. Additional antibodies included anti-transglutaminase IgA (anti-TTg IgA) and antinuclear antibodies (ANA). However, none of the individuals showed evidence of inflammation, as assessed by C-reactive protein tests and erythrocyte sedimentation rate evaluations^12^ . 

 The patients in the study by Cardinale et al.^12^ were treated with intravenous immunoglobulin (IVIg), in addition to other therapeutic options such as the monoclonal antibody rituximab and the immunosuppressant mycophenolate mofetil (MMF). All patients showed improvements in overall function and common manifestations of DSRD following immunotherapy. According to the authors, these improvements were achieved more rapidly compared to the natural course of the disease, which typically involves a slow and incomplete recovery. 

 Similar to the findings of Cardinale et al.^12^ , Worley et al.^10^ , in a case-control study, reported an association between DSRD and thyroid autoimmunity, suggesting an autoimmune etiology for the disorder. In the groups evaluated, the seropositivity rate for anti-TPO was higher in the cases (10/11; 91%) compared to the controls (5/21; 23%). Among the seropositive patients, eight were treated for hypothyroidism and one for Graves’ disease, and all remained euthyroid during treatment. 

 The study conducted by Santoro et al.^5^ also suggested a chronic autoimmune etiology for DSRD. This research was a multicenter, non-randomized, prospective observational study that included 82 individuals with DSRD, of both sexes, aged between 8 and 26 years at symptom onset. Patients were treated with high-concentration IVIg (10%, 100 mg/dL), with maintenance doses ad ministered every 28±3 days, followed by a standardized tapering protocol after 9–12 months of treatment. 

 Clinical responses were favorable in approximately 85% of patients, with the most notable improvements observed in individuals with catatonia or abnormalities in neurodiagnostic studies (electroencephalogram, magnetic resonance imaging, lumbar puncture). About 46% of patients experienced neurological relapse following the tapering of IVIg, with those having a history of autoimmunity or neurodiagnostic abnormalities showing a higher likelihood of relapse. According to the authors, this suggests the potential for a chronic autoimmune etiology in some cases of DSRD^5^ . 

 In another study by Santoro et al.^19^ , although the primary aim was not to discuss the causes of DSRD, the findings on autoimmune disease and serum cytokines are noteworthy. In this retrospective study of 266 individuals with DS, both with and without DSRD, there was a significant difference in the history of autoimmune disease (38% in DSRD vs. 15% in non-DSRD, p<0.001) and the presence of serum cytokine abnormalities (29% in DSRD vs. 4% in non-DSRD, p<0.001). 

 Bonne et al.^2^ reported a series of four DSRD cases, involving two men and two women aged between 20 and 24 years. All four individuals exhibited altered mental status, cognitive decline, loss of previously acquired developmental milestones, circadian rhythm disturbances, and language deficits. All patients tested positive for autoimmune markers, with one showing hypergammaglobulinemia and three testing positive for thyroid autoantibodies–anti-TPO, anti-thyroglobulin (anti-TG), and anti-thyroid receptor. The cases responded, at least partially or temporarily, to treatment with corticosteroids and anti-inflammatories. Based on these results, the authors concluded that in some cases of DSRD, an autoimmune or inflammatory etiology seems plausible. 

 Although several studies point to an autoimmune etiology, DSRD appears to have other immunologically mediated causes that remain unclear^15^ . It is known that individuals with DS exhibit proteomic differences related to chronic immune dysregulation. In a case report published by Hart et al.^15^ , an 8-year-old girl diagnosed with DSRD, who showed no evidence of autoimmunity, almost completely returned to her premorbid status after immunotherapy treatment. 

 The patient studied by Hart et al.^15^ was tested for anti-TPO, anti-thyroglobulin, ANA, anti-beta-2-glycoprotein 1, and anti-cardiolipin antibodies, as well as for lupus anticoagulant and autoimmune encephalopathy panels, with all results considered normal. The immunotherapy treatment included a combination of IVIg, steroids (methylprednisolone), and the immunosuppressant mycophenolate mofetil (MMF). The patient exhibited a mild decline as the monthly administration of IVIg and steroids approached but responded with almost complete symptom resolution, except for persistent tics. According to the authors, this case demonstrates the therapeutic effect of IVIg and suggests underlying immune dysregulation. 

 Similarly, Garcia and Litra^17^ , in their case report, identified immune dysregulation as the cause of DSRD. The report describes the case of a 17-year-old girl with a history of congenital hypothyroidism and celiac disease who experienced significant weight loss (approximately 8 kg), altered mental status (lack of interest, non-verbal behavior), and loss of functional abilities within a 1-month period. The patient tested positive for anti-TPO and thyroid-stimulating immunoglobulin (TSI). The authors state that, following extensive investigation of journals, research, and case studies, and considering the diagnostic and laboratory tests, the final diagnosis of DSRD was attributed to immune dysregulation. They also noted that changes in medication and the return to high school acted as triggers for the development of DSRD in this patient. 

 In line with the previously discussed causes, Santoro et al.^3^ suggested that DSRD may have a neuroimmunological and neuroinflammatory origin in some cases. The authors conducted a multicenter case-control study, including 1,289 individuals with DS (72 diagnosed with DSRD and 1,217 without DSRD). The case group had a higher likelihood of a history of autoimmune disease and thyroid disease, as well as a significantly higher percentage of individuals testing positive for ANA and anti-TPO antibodies (13 and 37%, respectively) compared to the control group (5% for ANA and 23% for anti-TPO). The cytokine profile analysis also revealed that abnormalities were more prevalent in the case group (40%) than in the control group (13%), with elevated levels of soluble IL-2 receptor and IL-10 being the most frequent alterations. 

 The genetic contribution to the development of DSRD was recently investigated by Jafarpour et al.^20^ . Through exome sequencing, the authors identified de novo variants in genes involved in immune regulation in 20% of individuals with DSRD, with three likely pathogenic variants in the *DNASE1L3*, *UNC13D*, and *XIAP* genes, and one pathogenic variant in the *RNASEH2A* gene. Notably, individuals with these variants exhibited faster clinical decline, a higher rate of abnormalities on magnetic resonance imaging, and a greater likelihood of preceding triggers compared to individuals with DSRD who did not have these variants. 

 Building on these findings, Jafarpour et al.^20^ emphasize that the four mentioned genes encode proteins associated with the type 1 interferon inflammatory response. DS is linked to an amplified interferon response, with chromosome 21 carrying the genes encoding four of the six subunits of the interferon receptor. The presence of an extra copy of chromosome 21 increases the expression of interferon receptors, which, in turn, enhances the JAK/STAT signaling pathway, leading to upregulation of interferon-stimulated gene (ISG) expression in response to various triggers in individuals with DS. The authors suggest that variants in these genes may exacerbate the inflammatory response to multiple triggers, contributing to the development of DSRD. This hypothesis is supported by the observationof a higher likelihood of preceding triggers and faster clinical decline among individuals with these variants. 

 The role of stressors in the development of DSRD has also been investigated, with reports indicating their occurrence prior to symptom onset in 29 to 100% of cases^18^ . These stressors, referred to as psychosocial or environmental stressors, are considered contributing factors or triggers for DSRD^16 ,18^. A variety of stressors have been reported, including adolescence transition, medical hospitalization, changes in the home environment (relocation, divorce, illness, death), changes in the school environment (changing schools, new teacher), violence (psychological, physical, sexual), and substance abuse (alcohol or drugs)^11^ . 

 According to Walpert et al.^14^ , DSRD may be better understood as a stress-triggered condition that occurs in individuals with DS who have an additional genetic factor or an acquired vulnerability. Other authors suggest that the interaction between stressors and a predisposition to immune dysregulation in the DS population could unify the overlap between the potential neuroimmunological and psychiatric origins of DSRD through the concept of the neuroendocrine system, although the exact pathogenic mechanism remains unknown^18^ . 

 In the study by Wang et al.^18^ , 337 individuals with DS, both with and without a diagnosis of DSRD, were evaluated for the occurrence of adverse childhood experiences (ACEs). It was observed that there was no significant difference in the prevalence of ACEs between individuals with and without DSRD (p=0.18). However, among participants with the disorder, the report of three or more ACEs (33%) was higher than in participants without the diagnosis (22%). Based on these results, it was considered that the presence of ACEs is not sufficient to explain the development of DSRD, and it remains unclear whether they are predictive of the condition. Nonetheless, it was observed that immunotherapy was less effective in patients with multiple ACEs occurring 3 months prior to symptom onset, which may suggest a more strongly psychiatric etiology in these cases. 

 In a 2020 case-control study, Santoro et al.^13^ observed a significant difference in the prevalence of specific stressors between individuals with DSRD and those without. The study included 35 cases and 35 controls matched by sex, age, and ethnicity. The cases experienced an average of 1.1±1.1 stressors, while the controls had 0.2±0.4 stressors (p<0.001). A higher prevalence of stressors in the case group was noted, particularly for events such as changing schools or jobs and the disruption of relationships with a family member, friend, or caregiver. According to the authors, these findings suggest that psychological factors may play a role in DSRD and could precede the onset of symptoms. 

 Similarly, in the 2023 study by Santoro et al.^19^ , which included 266 individuals with and without DSRD, a significant difference in the occurrence of a preceding trigger was observed between the two groups (48% in DSRD and 19% in non-DSRD). The most frequently reported events were infections (52%) and changes in the home/school/work environment (16%). In another study by Santoro et al.^3^ , which included 72 individuals with DSRD, a potential preceding trigger was identified in 51% of cases. The most frequently reported events in this study aligned with the previous one, with recent infections reported in 43% of individuals and changes in the home/school/work environment in 27%. 

 In the study by Mircher et al.^11^ , the presence of a stressor was also evident in the majority of DSRD cases. The authors retrospectively evaluated the cohort from the Jerome Lejeune Institute (JLI), which included 6,000 individuals with DS, aged 0–75 years, with up to 60 years of follow-up. Notably, 30 cases of DSRD were identified, with regression typically starting around 20 years of age, and 64% of the individuals were female. In 50% of the participants, the regression coincided with either a school change or parental separation. For 23% of cases, the event was related to the patient’s awareness of their condition or a sibling’s marriage. Other reported conditions included assault (17%), illness or death of a family member or friend (13%), and overstimulation caused by parental academic or vocational expectations (10%). According to the authors, a significant stress event in adolescents with DS may lead to a dramatic reduction in neurotransmitters, which in turn could contribute to the onset of acute regression. 

 In the study conducted by Sargado et al.^16^ , all evaluated individuals experienced at least one, and often several, psychosocial stressors in the year preceding the regression. This research included 14 individuals with DSRD, aged 7–20 years, with eight females and six males. Among the participants, 12 experienced two or more stressors, and one individual encountered stressors in all the considered categories, namely traumatic events, adolescence transition, changes at home, changes at school, and changes in social relationships. The most commonly observed event was changing to a new school, followed by abuse/highly stressful events and puberty-related changes/bullying. In the systematic review by Walpert et al.^14^ , which included 13 studies, the category of events termed transition/change in environment was the most frequently reported. Within this category, the most prevalent events were school completion/dropout and moving residence. 

 The assessment of the studies using the JBI Critical Appraisal Tools^9^ generally revealed that the included studies were considered methodologically adequate, although Walpert et al.^14^ and Santoro et al.^5^ presented limitations. Walpert et al.^14^ showed inconsistencies in the research question and methodology, including unclear duplicate screening and the absence of a third reviewer or publication bias assessment. Santoro et al.^5^ lacked clarity on participant follow-up duration and did not adequately control for confounding factors related to disease severity. While these issues do not significantly impact the interpretation of findings, they highlight potential weaknesses in the quality of the evidence. 

 Regarding the limitations of this study, it is important to acknowledge that the search was restricted to four bibliographic databases. Additionally, to enhance clarity, assessment of the quality of the included sources of evidence was conducted. Given that DSRD is a rare condition still in the process of characterization and knowledge dissemination, the inclusion of small sample sizes was expected and is not considered a factor that compromises the quality of the studies evaluated. Despite efforts to develop a comprehensive search strategy, the selection of terms may have been limited by the fact that this is still a relatively unknown topic with emerging and scarce literature. The recent definition of the nomenclature for DSRD may have also contributed to retrieving fewer studies. Moreover, heterogeneity among the included studies, particularly in terms of the number of participants and the diagnostic tests performed, limits the generalizability of the results. Finally, the challenge of proving causality in DSRD must be acknowledged, especially given the lack of sufficient longitudinal data. 

 Based on the evidence presented by the studies included in this scoping review, the hypothesis that DSRD is a multifactorial condition seems reasonable. Nevertheless, the immune system may play a key role in its development, as the identified causes converge toward a neuroinflammatory process. Several studies point to chronic autoimmunity and immune dysregulation as causes, based on the findings of autoantibodies and cytokine alterations, as well as the therapeutic response to immunotherapy. Furthermore, a neuroimmunological and neuroinflammatory etiology was suggested in one of the studies. 

 The contribution of genes to the manifestation of DSRD also seems plausible, as genetic variants associated with the type 1 interferon inflammatory response were identified in individuals with DSRD, suggesting that such variants may exacerbate the inflammatory response to various triggers. Finally, the role of psychosocial or environmental stressors was highlighted, as these are considered potential triggers in the development of DSRD. The studies report the occurrence of stress events, such as the transition through puberty and changes in home and school environments, in most cases of DSRD, preceding the onset of symptoms. 

 Given the still limited understanding of the causes and factors contributing to the development of DSRD and considering the limitations of the studies conducted so far, it is evident that further longitudinal research with a larger number of participants is needed to investigate more deeply the proposed immunological and neuroinflammatory mechanisms. Similarly, the causality of the identified genetic variants requires validation through functional studies and their application on a larger scale, considering the influence of environmental factors at the interface between genotype and phenotype. It is also essential to enhance the understanding of the role of stress events and to evaluate the use of potential biomarkers that could help elucidate the etiology of DSRD. 

## Data Availability

No new data were generated or analyzed in this study.
