# Supplementary material for: Causes of Down syndrome regression disorder: a scoping review
Source: Dement Neuropsychol. 2025 Jul 18;19(Suppl 1):e20240233. doi: 10.1590/1980-5764-DN-2024-0233 (PMC12306486; doi:10.1590/1980-5764-DN-2024-0233)
Supplement: Supplementary Material [file 1980-5764-dn-19-s1-e20240233-suppl01.docx]

**Table. Risk of bias**

| **N** | **Year** | **Title** | **Authorship** | **Objective** | **Study type** | **Number of participants** | **Risk of bias** |
| --- | --- | --- | --- | --- | --- | --- | --- |
| 1 | 2015 | Down syndrome disintegrative disorder: new-onset autistic regression, dementia, and insomnia in older children and adolescents with Down syndrome. | Worley et al.^10^ | To investigate the occurrence of new-onset autistic regression, dementia and insomnia in children and adolescents with Down syndrome. | Case control | 11 (DSRD) and 21 (non-DSRD) | Adequate. Include |
| 2 | 2017 | Acute regression in young people with Down syndrome | Mircher, et al.^11^ | To share the authors' experience with the report of 30 cases of regression in young DS who were followed longitudinally. | Cohort | 30 | Adequate. Include |
| 3 | 2019 | Immunotherapy in selected patients with Down syndrome disintegrative disorder | Cardinale et al.^12^ | Evaluate the response to immunotherapy in a small cohort of patients with DSRD who had evidence of autoimmunity. | Case series | 4 | Adequate. Include |
| 4 | 2020 | Unexplained regression in Down syndrome: 35 cases from an international Down syndrome database | Santoro et al.^13^ | Describe cases of DSRD and compare them with age- and sex-matched Down syndrome patients. | Case control | 35 (DSRD) and 35 (non-DSRD) | Adequate. Include |
| 5 | 2021 | A systematic review of unexplained early regression in adolescents and adults with Down syndrome | Walpert et al.^14^ | Identify patterns of symptomatology, potential trigger events, prognosis, treatments, and outcomes surrounding unexplained regression in adolescents and young adults with Down syndrome. | Systematic review | Not applicable | Adequate.  Revised with observations^a^ |
| 6 | 2021 | Case report: improvement following immunotherapy in an individual with seronegative Down syndrome disintegrative disorder | Hart et al.^15^ | Present the case of an 8-year-old girl with DSRD without evidence of autoimmunity. | Case report | 1 | Adequate. Include |
| 7 | 2022 | Is developmental regression in Down syndrome linked to life stressors? | Sargado et al.^16^ | Understand the role of psychosocial stressors in DSRD. | Case series | 14 | Adequate. Include |
| 8 | 2022 | Evidence of neuroinflammation and immunotherapy responsiveness in individuals with down syndrome regression disorder | Santoro et al.^3^ | Investigate the potential role of neurologic and neuroimmunologic dysfunction in persons with DSRD and whether the presence of abnormalities dictates response to particular therapeutic interventions. | Case control | 72 (DSRD) and 1217 (non-DSRD) | Adequate. Include |
| 9 | 2022 | Abnormal weight loss in an adolescent female with Down syndrome | Garcia et al.^17^ | Report the case of a patient with Down syndrome who exhibited weight loss, altered mental status, and loss of functional skills over a period of one month. | Case report | 1 | Adequate. Include |
| 10 | 2023 | Immunotherapy responsiveness and risk of relapse in Down syndrome regression disorder | Santoro et al.^5^ | Investigate possible demographic, laboratory, and clinical factors associated with the response to IVIg immunotherapy and to assess the likelihood of gradually tapering the treatment successfully once symptom improvement has been achieved. | Cohort | 82 | Adequate.  Revised with observations^b^ |
| 11 | 2023 | Adverse childhood experiences and the development of Down syndrome regression disorder | Wang et al.^18^ | Evaluate whether adverse childhood experiences (ACEs) were more prevalent in children with DRSD than in those with Down syndrome alone. | Case control | 159 (DSRD) and 178 (non-DSRD) | Adequate. Include |
| 12 | 2023 | Down syndrome regression disorder, a case series: clinical characterization and therapeutic approaches | Bonne et al.^2^ | Describe the DSRD, discuss its etiologies and propose therapeutic strategies. | Case series | 4 | Adequate. Include |
| 13 | 2023 | Alternative diagnoses in the work up of Down syndrome regression disorder. | Santoro et al.^19^ | Review non-DSRD diagnoses at a quaternary medical center specializing in the diagnosis of DSRD and compare clinical characteristics between those diagnosed with DSRD and those with non-DSRD diagnoses. | Case control | 212 (DSRD) and 54 (non-DSRD) | Adequate. Include |
| 14 | 2024 | De novo variants in immune regulatory genes in Down syndrome regression disorder | Jafarpour et al.^20^ | Evaluate contribution of rare variants within coding regions of genes related to immune regulation to DSRD. | Cohort | 41 (DSRD with exome sequencing) and 306 (DSRD without exome sequencing) | Adequate. Include |

1. The systematic review conducted by **Walpert et al. (2021)** highlighted inconsistencies related to the formulation of the research question and methodological aspects. Notably, there was a lack of clarity in the process of duplicate screening and the involvement of a third reviewer, as well as the absence of an assessment of publication bias in the included studies.
2. The cohort study by **Santoro et al. (2023)** presented uncertainties regarding follow-up duration, as it was not reported whether all individuals remained in the study until its completion, nor were any potential sample losses described. Additionally, possible confounding factors related to the severity of the participants’ clinical condition were not adequately controlled and were only mentioned as study limitations.
